# Digestibility and Quality Characteristics of *Sulgidduk* (a Traditional Korean Rice Cake) Prepared with Malic Acid-Treated Wheat Starch

**DOI:** 10.4014/jmb.2505.03037

**Published:** 2025-06-18

**Authors:** Gyeong A Jeong, Inae Lee, Chang Joo Lee

**Affiliations:** 1 Department of Food Science and Biotechnology, Wonkwang University, Iksan 54538, Republic of Korea; 2 Department of Food Science and Biotechnology, Kyung Hee University, Yongin 17104, Republic of Korea

**Keywords:** Resistant starch, malic acid, wheat starch, *Sulgidduk*, rice flour, flour substitute

## Abstract

With rising obesity rates worldwide and growing health awareness, the demand for low-calorie, blood sugar-controlling products is heightening. Particularly, research has increasingly explored the incorporation of resistant starch (RS) in dietary applications, as it does not add to calorie intake and helps regulate blood sugar levels, prevent constipation, and increase fecal volume, similar to dietary fiber. This study evaluated the quality characteristics and digestibility of malic acid-treated wheat starch (MA starch) with a high RS content for use as a rice flour substitute in *Sulgidduk*. MA starch was incorporated at 10–40% of the rice flour weight. Adding MA starch to *Sulgidduk* resulted in slight color changes, with decreased lightness and increased redness and yellowness, although structurally it remained unchanged. Higher MA levels reduced the pH and moisture content but increased sugar content and hardness. Compared to the control sample (rapidly digestible starch (RDS): 68.3%, RS: 30.3%), *Sulgidduk* prepared with MA starch had lower RDS (55.7%) and higher RS (42.3%) content. MA starch significantly affected RDS and RS levels, which are associated with the glycemic index, while also affecting moisture content and textural characteristics. Based on these findings, 30% substitution with MA starch (MA-30) appears to be the optimal ratio for maintaining health benefits while minimizing quality degradation. Collectively, these results highlight the potential of MA starch as a functional food ingredient and support its application in the formulation of dietary products for calorie management.

## Introduction

According to the World Health Organization (WHO), approximately 890 million people worldwide are classified as obese, and obesity poses significant health risks, including hypertension, heart disease, cancer, stroke, and psychological disorders [1]. Although various environmental and physiological factors contribute to obesity, dietary habits play a central role in weight gain, underscoring their significance in obesity prevention and management [2, 3]. Obesity is caused by the excessive consumption of obesogenic foods such as high-fat, high-calorie foods, and related medical expenses exceed $1,900 per individual annually [4, 5]. Accordingly, novel food products designed to indirectly improve health are being developed to align with evolving eating habits that prioritize nutrition and well-being [6, 7].

Carbohydrates serve as the primary energy source for the human body; however, excessive carbohydrate intake contributes to obesity owing to increased caloric consumption [8, 9]. Starch, a primary dietary carbohydrate, is categorized as either rapidly digestible starch (RDS), slowly digestible starch (SDS), or resistant starch (RS) based on its digestion characteristics [10, 11]. These carbohydrates significantly influence the glycemic index and play a crucial role in the management and prevention of type 2 diabetes [12]. RDS is rapidly converted into glucose within 20 min of ingestion, sharply increasing blood sugar levels [13]. In contrast, SDS is digested more gradually between 20 and 120 min post-ingestion, whereas RS remains undigested in the small intestine beyond 120 min and is fermented by the gut microbiota in the large intestine. In contrast to digestible starches, RS does not contribute to calorie intake but helps regulate blood sugar levels [14-16]. Additionally, RS functions similarly to dietary fiber by preventing constipation and increasing fecal volume [17, 18]. RS fermentation produces short-chain fatty acids (*e.g.*, acetic, butyric, and propionic acids), which exhibit anticancer properties, particularly for the prevention of colon cancer [18, 19]. Consequently, increasing research is focusing on incorporating RS as a low-calorie starch alternative to address high-calorie dietary patterns [20-22]. RS is classified into five types: RS1, which is physically trapped starch; RS2, consisting of amylopectin crystals within native granular starch; RS3, formed by amylose crystals in retrograded starch; RS4, produced through chemical modification; and RS5, which is lipid-complexed starch [23]. Notably, RS4 exhibits superior heat stability and higher RS content than the other types [24, 25]. Our previous research successfully developed a modified starch with an RS content of 99.5% and confirmed its excellent heat stability, highlighting its potential as a functional ingredient in processed foods [26].

Rice cakes are a traditional food primarily made from rice that is popular in Asia [27]. *Dduk* (also known as "Korean rice cake") is available in various shapes, colors, and types, depending on the preparation method, which includes steaming, coating, or fermentation [28]. Among these, *Sulgidduk* (also called *Backsulgies*) is the most basic form and is prepared by steaming rice flour. Several studies have explored the enhancement of the quality and physiological properties of rice cakes by incorporating functional ingredients [27-29], whereas others have investigated the impact of modified rice flour and starch on their physical and storage properties [30-32]. However, few studies have investigated the digestibility and quality characteristics of rice cakes prepared using modified starch as a rice flour substitute for calorie control and dietary improvement. Therefore, this study aimed to evaluate the effects of substituting rice flour with RS-enriched modified starch in rice cake production. Specifically, malic acid-treated wheat starch (MA starch) was used to replace 10–40% of rice flour in *Sulgidduk* preparation. The color, pH, moisture and sugar content, texture, and starch digestibility characteristics of *Sulgidduk* prepared with MA starch were analyzed to determine the optimal formulation that enhances digestibility while minimizing quality degradation.

## Materials and Methods

### Materials

Rice flour (Injekirin Nonghyup Co. Ltd., Republic of Korea), wheat starch (Roquette, France), sugar (Beksul, CJ Cheil Jedang, Incheon, South Korea), and salt (Hanju Co., Republic of Korea) were purchased from a local grocery market. DL-malic acid (M1210) and porcine pancreatin (P7545; activity: 8× United States Pharmacopeia [USP]/g) were purchased from Sigma-Aldrich (USA). Amyloglucosidase (AMG 300 L, activity: 300 amyloglucosidase units [AGU]/ml) was obtained from Novozymes, Inc. (Denmark). A glucose oxidase-peroxidase (GOD–POD) kit was acquired from Asan Pharm Co., Ltd. (Republic of Korea).

### Preparation of MA Starch

MA starch was prepared according to the malic acid and heat treatment method described by Mansur *et al*.,[26]. Wheat starch was mixed with 4 M malic acid (pH 1.2) in a 1:1 ratio and soaked in water at room temperature for 16 h. The mixture was then dried to a moisture content below 10% using a 45°C hot air dryer (C-DF3, Changshin Science, Republic of Korea). The dried product was ground, heat-treated at 130°C for 7 h, and washed with distilled water and 95% ethanol to remove residual malic acid. After drying at 45°C, the final product was passed through a 150-mesh standard sieve (No. 150; Chunggye, Republic of Korea). The RS content of the prepared MA starch was confirmed to be 99.5%.

### Preparation of *Sulgidduk* with MA Starch

The mixing ratios of *Sulgidduk* prepared with MA starch are listed in Table 1. Mixtures of MA starch and rice flour were prepared by substituting rice flour with MA starch at concentrations of 10% w/w (MA-10), 20% (MA-20), 30% (MA-30), and 40% (MA-40). To the mixed powder, 10% sugar, 1% salt, and 40% moisture were added, followed by thorough mixing and sieving through a 200-mesh sieve. Control *Sulgidduk* was prepared using rice flour, sugar, salt, and water. A 45 g portion of the prepared powder was placed in a stainless-steel cylindrical mold (diameter: 5 cm, length: 4.5 cm), lined with cotton cloth, and steamed in a steamer (RCO-240CE, Rinnikorea Co., Republic of Korea). The top of the mixture was flattened and covered with dry cotton cloth to prevent condensation during steaming. After steaming for 30 min, the steamer was turned off, and the mixture was left to rest for 10 min. After cooling for 30 min at room temperature, the quality characteristics and digestibility of the *Sulgidduk* samples (control and MA starch-prepared) were analyzed.

### Appearance and Color Measurement

The *Sulgidduk* samples were placed in a petri dish (50 × 15 mm), and their color attributes, *L* value (lightness), a value (redness), and b value (yellowness), were measured thrice using a Hunter colorimeter (CM-5, Minolta Co., Japan) with a 30 mm diameter aperture. Additionally, the color difference (Δ*E*) was calculated using the following equation:

$$\Delta E=\sqrt{L^{2}+a^{2}+b^{2}}$$

To evaluate the appearance of *Sulgidduk* prepared with MA starch, samples were placed on a black stainless-steel plate at regular intervals, and images were taken to observe differences in visual characteristics.

### Moisture Content Measurement

The moisture content of *Sulgidduk* samples (5 g) was determined using an infrared moisture content meter (MB 120, Ohaus Co., USA). Measurements were performed in triplicate for each experimental group, and the average values were used.

### pH and Sugar Content Measurement

The pH of the *Sulgidduk* samples was measured by adding 15 ml of distilled water to 5 g of the sample. The mixture was homogenized using a homogenizer (T18D, IKA, Germany), followed by centrifugation at 3,000 ×*g* for 40 min. The supernatant was analyzed using a pH meter (Orion Star A215; Thermo Fisher Scientific, USA). The sugar content was quantified using the same supernatant using a saccharometer (Refractometer PAL-1, Atago, Japan) and expressed as °Brix value.

### Texture Profile Analysis

The textural characteristics of the *Sulgidduk* samples were measured using a Texture Analyzer (TA-XT2, Stable Micro Systems, England). The analysis was conducted in Texture Profile Analysis (TPA) mode with a P/35-cylinder probe. Briefly, the *Sulgidduk* sample was placed on a plate and subjected to a two-cycle compression test to deform 70% of its total thickness. The hardness, springiness, cohesiveness, gumminess, chewiness, and adhesiveness were evaluated. The detailed measurement conditions are listed in Table 2.

### *In Vitro* Starch Digestibility

The digestibility of the *Sulgidduk* samples was determined based on the methods described by Mansur *et al*.,[26], Shin *et al*., [33] with slight modifications. Briefly, *Sulgidduk* samples were freeze-dried (LP10; Ilshin Biobase, Republic of Korea), ground in a mortar, and sieved through a 150-mesh sieve for further analysis. A starch digestive enzyme solution was prepared by mixing pancreatin and amyloglucosidase. A 30 mg sample of freeze-dried *Sulgidduk*, 0.75 ml of sodium acetate buffer (0.1 M, pH 5.2), 0.75 ml of the enzyme solution, and glass beads (type-4) were added to a 2-ml tube and incubated in a shaking incubator (VS-8480SF, Vision Scientific Co., Republic of Korea). After 20 and 240 min, respectively, the samples were heated at 110°C for 10 min to terminate the reaction, cooled to room temperature, and centrifuged. The supernatant was analyzed using a GOD–POD kit to measure the glucose content. The color change corresponding to the glucose levels was measured at 505 nm using a UV-Visible Spectrophotometer (UV-1800, Shimadzu Co., Japan). The enzyme-RS content was calculated using the program provided with the GOD–POD kit. Glucose released at 20 min represented RDS, whereas that obtained at 20–240 min corresponded to SDS. The RS remained unhydrolyzed even after 240 min of incubation.

### Statistical Analysis

All experimental data were analyzed using SPSS (version 23.0; SPSS Inc., USA), and an analysis of variance (ANOVA) was performed, followed by Duncan’s multiple range test. Values are expressed as the means ± standard deviation, and statistical significance was defined as *p* < 0.05.

## Results and Discussion

### Appearance and Color of *Sulgidduk* Prepared with MA Starch

**Fig. 1:**
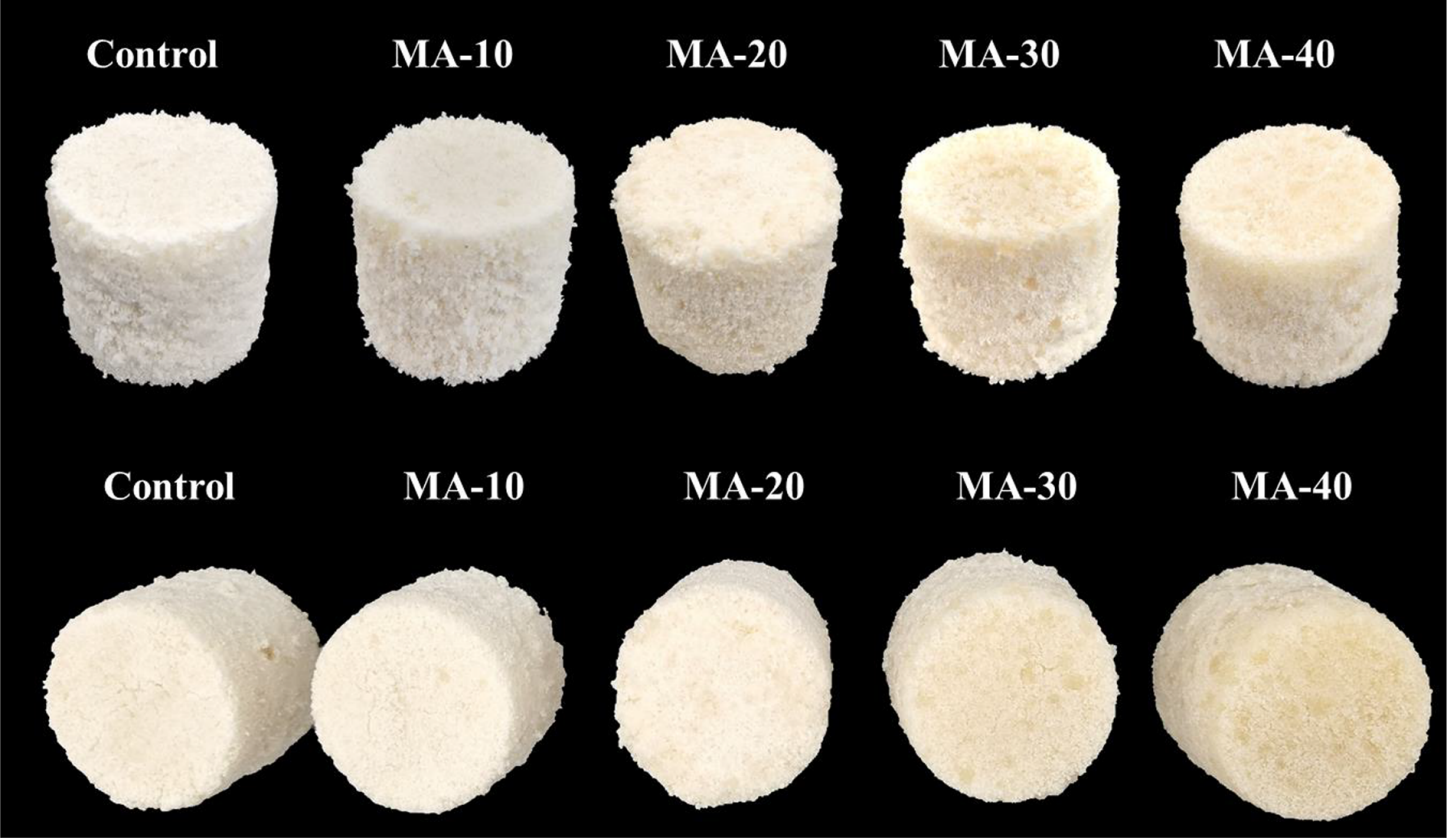
Appearance of *Sulgidduk* prepared with various proportions of malic acid-treated wheat starch (MA starch). Control: 100% rice flour, MA-10: rice flour replaced with 10% MA starch, MA-20: rice flour replaced with 20% MA starch, MA-30: rice flour replaced with 30% MA starch, MA-40: rice flour replaced with 40% MA starch.

The appearance and color of *Sulgidduk* samples are shown in Fig. 1 and Table 3. *Sulgidduk* is primarily composed of rice flour, giving it a naturally white appearance [27]. Similarly, the control sample, which contained only rice flour, exhibited a bright white color. However, as the proportion of MA starch increased, a noticeable increase in yellowness was noted. The lightness (*L*) value in the color analysis, representing whiteness, was highest in the control sample (89.5) and progressively decreased as the proportion of MA starch increased. Although MA starch is white in color, acid treatment enhances its reducing power through acid hydrolysis, leading to the production of low molecular-weight sugar compounds [34]. Exposure to high temperatures during rice cake processing promotes the Maillard reaction, in which these sugar molecules reduce whiteness while increasing both redness and yellowness [35, 36]. Consistent with this, as the MA starch content increased, the *L* value decreased from 87.3 (MA-10) to 83.7 (MA-40), while redness increased from 0.28 (MA-10) to 0.81 (MA-40) and yellowness increased from 9.76 (MA-10) to 14.3 (MA-40). Moreover, rice flour substitutes and additives in rice cake formulations can affect the structural stability of the final product. In this study, the structural integrity of *Sulgidduk* remained intact even after the incorporation of MA starch, and its overall shape closely resembled that of the control sample. These findings suggested that MA starch can serve as a suitable additive for *Sulgidduk*.

### Moisture Content, pH, and Sugar Content of *Sulgidduk* Prepared with MA Starch

The moisture, pH, and sugar content of *Sulgidduk* samples are listed in Table 4. As the amount of added MA starch increased, the moisture content decreased compared with that of the control sample (32.9%), with values ranging between 32.7% and 29.9%. RS4-type modified starch undergoes hilum region degradation owing to acid treatment, which destroys its crystalline structure and reduces both its viscosity and swelling power [16]. This decrease in viscosity and swelling power appears to reduce the moisture absorption capacity of the modified starch, resulting in lower moisture content as its concentration increases. The pH of *Sulgidduk* samples also decreased significantly (*p* < 0.05) from 6.24 (Control) to 2.77 (MA-40) as the amount of MA starch increased. This reduction in pH was likely attributed to the acidic nature of the malic acid treatment solution used in the starch modification process, which had a pH of 1.2. This aligns with previous studies that have reported that RS4-type modified starches produced using organic acids exhibit a low pH [37]. Additionally, the sugar content of *Sulgidduk* samples increased as the proportion of MA starch increased, with °Brix values rising from 1.83 (Control) to 3.30 (MA-40). This increase in the °Brix value was likely due to the presence of low molecular weight sugar compounds generated during the malic acid treatment process [34].

### Texture of *Sulgidduk* Prepared with MA Starch

Textural analysis of *Sulgidduk* samples is presented in Table 5. Hardness in the control sample was measured at 54.1 N, whereas that in *Sulgidduk* samples prepared with MA starch increased from 70.3 N to 101 N as the proportion of MA starch increased. This increase in the hardness of *Sulgidduk* prepared with MA starch is likely attributable to the reduction in moisture content, which decreases the overall water retention of *Sulgidduk*, leading to a firmer texture. The decrease in moisture content also influenced other textural properties, including springiness, cohesiveness, gumminess, chewiness, and adhesiveness, all of which increased with increasing MA starch concentrations. Previous studies have demonstrated that the addition of powders during *Sulgidduk* processing generally increases textural firmness compared to that of the control without additives [27, 38, 39], with the moisture absorption capacity of the additive playing a key role. Given these findings, it is necessary to establish the maximum amount of MA starch to be added. Based on the textural analysis, the MA-10, MA-20, and MA-30 formulations, which exhibited cohesiveness, gumminess, springiness, and chewiness characteristics most similar to those of the control sample, appeared to be the most suitable for preparing *Sulgidduk* with MA starch.

### Digestibility of *Sulgidduk* Prepared with MA Starch

The digestibility of *Sulgidduk* samples is listed in Table 6. The digestibility analysis revealed that the control sample contained 68.3% RDS, 1.39% SDS, and 30.3% RS. In *Sulgidduk* samples containing MA starch, the SDS values did not exhibit significant differences (*p* > 0.05). However, both the RDS and RS contents were significantly changed with the addition of MA starch, with RDS content decreasing to 65.3% (MA-10), 63.4% (MA-20), 61.1%(MA-30), and 55.7% (MA-40), while the RS content increased to 33.1% (MA-10), 34.9% (MA-20), 37.8% (MA-30), and 42.3% (MA-40). Commercially available wheat starch naturally contains SDS and RS (types 1 and 2); however, owing to its low thermal stability, it is converted to RDS during cooking [40, 41]. To address this issue, RS with enhanced heat stability has been developed through physical, chemical, and enzymatic modification methods [42]. The modified starch used in this present study was chemically modified using malic acid, resulting in strong cross-linking that enhanced thermal stability, maintained RS characteristics after cooking, and achieved a high RS content of up to 99.5% [26]. Products containing such modified starches aid in glycemic index control and offer health benefits associated with low-calorie diets [14].

In this study, increasing the proportion of MA starch in *Sulgidduk* led to a higher RS content while reducing RDS levels, which influence the glycemic index. This suggests that *Sulgidduk* prepared with MA starch may help mitigate rapid increases in blood glucose levels compared with the control *Sulgidduk*. Although MA-40 exhibited the most significant potential health benefits, its excessive addition resulted in a reduced moisture content and undesirable textural deterioration. Therefore, MA-30, which showed the highest RS content while maintaining quality characteristics similar to those of the control sample, was considered the optimal formulation for *Sulgidduk*.

## Conclusion

This study investigated the quality characteristics and digestibility changes in *Sulgidduk*, a traditional Korean rice cake, by partially substituting rice flour with 10%, 20%, 30%, and 40% (w/w) MA starch, which has high RS content and excellent thermal stability. The structural integrity of *Sulgidduk* was maintained, demonstrating its suitability as an additive. However, as MA starch was added, lightness decreased, and redness and yellowness increased compared to the control sample. Furthermore, the pH and moisture content of *Sulgidduk* prepared with MA starch decreased with increasing amounts of MA starch, whereas sweetness levels increased. Texture analysis revealed that hardness, springiness, cohesiveness, gumminess, chewiness, and adhesiveness increased owing to the reduction in moisture content. Moreover, as the proportion of MA starch increased, the RDS content decreased, whereas the RS content peaked at 42.3% (MA-40). However, considering the overall quality characteristics, the MA-30 formulation, which exhibited a texture comparable to that of the control sample while maintaining high RS content, appeared to be the optimal mixing ratio for *Sulgidduk*. Taken together, this study highlighted the potential of MA starch, characterized by its high RS content and exceptional thermal stability, as a carbohydrate substitute for rice cake products. These findings confirm that the functional properties of MA starch were preserved even after processing, highlighting its broad applicability as a functional ingredient. Furthermore. MA starch is expected to be useful not only in rice cakes but also as an additive in various carbohydrate-based foods and dietary products for calorie control.

## Figures and Tables

**Table 1:** Formulas for *Sulgidduk* prepared with varying proportions of MA starch.

Sample	Ingredients (g)				
	Rice flour	MA starch	Sugar	Salt	Water
Control	100	-	10	1.00	40
MA-10	90	10	10	1.00	40
MA-20	80	20	10	1.00	40
MA-30	70	30	10	1.00	40
MA-40	60	40	10	1.00	40

**Table 2:** Texture analyzer operating conditions for *Sulgidduk* prepared with varying proportions of MA starch.

Item	Condition
Test type	TPA test
Measurement type	Two bite compression
Sample size	4.0 × 5.0 cm
Probe	35 mm dia, circle
Test speed	5.0 mm/sec
Deformation	70%
Trigger force	0.049 N

**Table 3:** Hunter’s color values of *Sulgidduk* prepared with varying proportions of MA starch.

Sample	Hunter’s color value			
	*L*	*a*	*b*	Δ*E*
Control	89.5 ± 0.13^e^	-0.17 ± 0.01^a^	8.80 ± 0.15^a^	89.9 ± 0.12^e^
MA-10	87.3 ± 0.11^d^	0.28 ± 0.00^b^	9.76 ± 0.05^b^	87.8 ± 0.11^d^
MA-20	85.3 ± 0.08^c^	0.66 ± 0.02^c^	12.2 ± 0.09^c^	86.2 ± 0.09^c^
MA-30	84.5 ± 0.49^b^	0.75 ± 0.03^d^	13.0 ± 0.12^d^	85.5 ± 0.50^b^
MA-40	83.7 ± 0.05^a^	0.81 ± 0.01^e^	14.3 ± 0.07^e^	84.9 ± 0.04^a^

^a-e^The values with different superscripts within a column are significantly different (*p* < 0.05) by Duncan’s multiple range test.

**Table 4:** Moisture content, pH, and °Brix values of *Sulgidduk* prepared with varying proportions of MA starch.

Sample	Moisture (%)	pH	°Brix
Control	32.9 ± 0.12^d^	6.24 ± 0.03^e^	1.83 ± 0.29^a^
MA-10	32.7 ± 0.11^d^	3.40 ± 0.06^d^	2.00 ± 0.17^ab^
MA-20	31.1 ± 0.20^c^	3.05 ± 0.02^c^	2.33 ± 0.23^bc^
MA-30	30.3 ± 0.04^b^	2.90 ± 0.05^b^	2.67 ± 0.25^c^
MA-40	29.9 ± 0.33^a^	2.77 ± 0.03^a^	3.30 ± 0.26^d^

^a-e^The values with different superscripts within a column are significantly different (*p* < 0.05) by Duncan’s multiple range test.

**Table 5:** Textural profiles of *Sulgidduk* prepared with varying proportions of MA starch.

Sample	TPA					
	Hardness (N)	Springiness	Cohesiveness	Gumminess (N)	Chewiness (N)	Adhesiveness (N.sec)
Control	54.1 ± 5.01^a^	0.185 ± 0.021^a^	0.093 ± 0.004^a^	6.53 ± 1.920^a^	1.56 ± 0.494^a^	-2.234 ± 0.448^a^
MA-10	70.3 ± 5.05^b^	0.236 ± 0.011^ab^	0.107 ± 0.006^b^	8.03 ± 0.160^ab^	2.06 ± 0.228^a^	-1.812 ± 0.327^b^
MA-20	75.1 ± 0.93^bc^	0.268 ± 0.015^bc^	0.116 ± 0.006^b^	8.65 ± 1.485^abc^	2.18 ± 0.211^a^	-1.618 ± 0.438^c^
MA-30	80.0 ± 2.07^c^	0.311 ± 0.037^cd^	0.129 ± 0.007^c^	9.91 ± 2.560^cd^	2.48 ± 0.337^a^	-1.354 ± 0.098^c^
MA-40	101 ± 4.00^d^	0.361 ± 0.052^d^	0.145 ± 0.006^d^	11.8 ± 1.457^d^	3.95 ± 1.009^b^	-1.153 ± 0.252^d^

^a-d^The values with different superscripts within a column are significantly different (*p* < 0.05) by Duncan’s multiple range test.

**Table 6:** Rapidly digestible starch (RDS), slowly digestible starch (SDS), and resistant starch (RS) yield (%) of *Sulgidduk* prepared with varying proportions of MA starch.

Sample	RDS (%)	SDS (%)	RS (%)
Control	68.3 ± 1.06^d^	1.39 ± 0.39^a^	30.3 ± 1.36^a^
MA-10	65.3 ± 1.53^c^	1.61 ± 0.93^a^	33.1 ± 1.56^b^
MA-20	63.4 ± 1.88^bc^	1.76 ± 1.02^a^	34.9 ± 1.38^b^
MA-30	61.1 ± 0.80^b^	1.12 ± 0.43^a^	37.8 ± 0.65^c^
MA-40	55.7 ± 1.02^a^	2.03 ± 0.39^a^	42.3 ± 0.63^d^

^a-d^The values with different superscripts within a column are significantly different (*p* < 0.05) by Duncan’s multiple range test.

## References

[1] (WHO) WHO. 2024. Obesity and overweight. Available from: https://www.who.int/en/news-room/fact-sheets/detail/obesity-andoverweight.Accessed Oct. 15, 2024.

[2] Albuquerque D, Nóbrega C, Manco L, Padez C (2017). The contribution of genetics and environment to obesity. Br. Med. Bull..

[3] Scott R, Tan T, Bloom S (2013). Gut hormones and obesity: physiology and therapies. Vitam. Horm..

[4] Kim DD, Basu A (2016). Estimating the medical care costs of obesity in the United States: systematic review, meta-analysis, and empirical analysis. Value Health.

[5] Reader SW, Lopez RB, Denny BT (2018). Cognitive reappraisal of low-calorie food predicts real-world craving and consumption of high-and low-calorie foods in daily life. Appetite.

[6] Sarteshnizi RA, Hosseini H, Bondarianzadeh D, Colmenero FJ (2015). Optimization of prebiotic sausage formulation: effect of using β-glucan and resistant starch by D-optimal mixture design approach. LWT Food Sci. Technol..

[7] Karunarathna S, Wickramasinghe I, Truong T, Brennan C, Navaratne S, Chandrapala J (2024). Development of low-calorie food products with resistant starch-rich sources.-a review. Food Rev. Int..

[8] Ai Y, Jane Jl. 2024. Chapter 3 - Understanding starch structure and functionality. pp. 55-77. *In* Nilsson L (eds.), *Starch in food*. 2024, 3rd ed. Woodhead Publishing.

[9] Van Dam R, Seidell J (2007). Carbohydrate intake and obesity. Eur. J. Clin. Nutr..

[10] Kaur L, Kaur R, Singh J. 2024. Chapter 5 - Chemical modification of starch. pp. 97-117. *In* Nilsson L (eds.), *Starch in food*. 2024, 3rd ed. Woodhead Publishing.

[11] Klostermann CE, Endika MF, Kouzounis D, Buwalda PL, de Vos P, Zoetendal EG, Bitter JH, Schols HA (2024). Presence of digestible starch impacts in vitro fermentation of resistant starch. Food Funct..

[12] Chisbert M, Castell AL, Vinoy S, Nazare JA (2024). The impact of slowly digestible and resistant starch on glucose homeostasis and insulin resistance. Curr. Opin. Clin. Nutr. Metab. Care.

[13] Nag S, Majumder S (2023). Starch, gallic acid, their inclusion complex and their effects in diabetes and other diseases-a review. Food Sci. Nutr..

[14] Bello-Perez LA, Hoyos-Leyva JD. 2018. Chapter 22 - Development of foods high in slowly digestible and resistant starch. pp. 827-854. *In* Sjöö M, Nilsson L (eds.), *Starch in food*. 2018, 2nd ed. Woodhead Publishing.

[15] Shimotoyodome A, Suzuki J, Fukuoka D, Tokimitsu I, Hase T (2010). RS4-type resistant starch prevents high-fat diet-induced obesity via increased hepatic fatty acid oxidation and decreased postprandial GIP in C57BL/6J mice. Am. J. Physiol. Endocrino.l Metab..

[16] Na JH, Jeong GA, Park HJ, Lee CJ (2021). Impact of esterification with malic acid on the structural characteristics and in vitro digestibilities of different starches. Int. J. Biol. Macromol..

[17] Nugent AP (2005). Health properties of resistant starch. Nutr. Bull..

[18] Fuentes-Zaragoza E, Riquelme-Navarrete M, Sánchez-Zapata E, Pérez-Álvarez J (2010). Resistant starch as functional ingredient: a review. Food Res. Int..

[19] Lockyer S, Nugent A (2017). Health effects of resistant starch. Nutr. Bull..

[20] Menon R, Padmaja G, Sajeev M (2015). Cooking behavior and starch digestibility of NUTRIOSE^®^(resistant starch) enriched noodles from sweet potato flour and starch. Food Chem..

[21] Tsatsaragkou K, Papantoniou M, Mandala I (2015). Rheological, physical, and sensory attributes of gluten‐free rice cakes containing resistant starch. J. Food Sci..

[22] Rojhani A, Naranjo J, Ouyang P (2022). Physiochemical properties and sensory characteristics of resistant starch enriched cookies. Nutr. Food Sci..

[23] Birt DF, Boylston T, Hendrich S, Jane J-L, Hollis J, Li L (2013). Resistant starch: promise for improving human health. Adv. Nutr..

[24] Dong H, Vasanthan T (2020). Amylase resistance of corn, faba bean, and field pea starches as influenced by three different phosphorylation (cross-linking) techniques. Food Hydrocoll..

[25] Wang S, Guo P. 2020. Botanical sources of starch. pp. 9-27. *In* Wang S (eds.), *Starch structure, functionality and application in foods*. 2020. Singapore: Springer Singapore.

[26] Mansur AR, Jeong GA, Lee CJ (2022). Preparation, physicochemical properties, and in vivo digestibility of thermostable resistant starch from malic acid-treated wheat starch. Food Res. Int..

[27] Zhang Y, Kim JH, Song KY, Kim YS (2016). Quality characteristics and antioxidant activities of *Sulgidduck* with asparagus (*Asparagus officinalis* L.) powder. J. East Asian Soc. Diet. Life.

[28] Choi EJ, Kim EK (2017). Effect of *Moringa oleifera* leaf on antioxidant and quality characteristics of the Korean traditional rice cake sulgidduk. J. Food Process. Preserv..

[29] Baek SY, Kim SJ, Kim MR. 2019. Physicochemical properties and antioxidant activities of *Sulgidduk* added with *Enteromorpha prolifera*. *J. Korean Soc. Food Sci. Nutr.* 10.3746/jkfn.2019.48.12.1373

[30] Park HJ, Song JC, Shin WC (2006). Optimization of modified starches on retrogradation of Korean rice cake (Garaeduk). Korean J. Food Nutr..

[31] Xiaohuang C, Qianqian H, Cong Y, Azam MS, Ahiduzzaman M, Islam MN (2022). Enhancement of the selected physico-chemical properties of steamed rice cake by the application of acetylated distarch adipate. J. Food Meas. Charact..

[32] Jung HJ, Choi HW, Kim BY, Baik MY (2017). Rheological properties of rice flour treated with mild solutions of citric acid. Food Sci. Biotechnol..

[33] Shin SI, Lee CJ, Kim DI, Lee HA, Cheong JJ, Chung KM (2007). Formation, characterization, and glucose response in mice to rice starch with low digestibility produced by citric acid treatment. J. Cereal Sci..

[34] Surendra Babu A, Parimalavalli R, Rudra SG (2015). Effect of citric acid concentration and hydrolysis time on physicochemical properties of sweet potato starches. Int. J. Biol. Macromol..

[35] Barua S, Srivastav P (2017). Effect of heat-moisture treatment on resistant starch functional and thermal properties of mung bean (Vigna radiate) starch. J. Nutr. Health Food Eng..

[36] Trithavisup K, Charoenrein S (2016). Influence of acid treatment on physicochemical properties of aged rice flour. Int. J. Food Prop..

[37] Song JY, No JH, Shin M (2016). Effects of resistant starch on the viscosity and stability of fat-free dressing. Korean J. Food Cook. Sci..

[38] Jun MK, Kim MY, Chun SS (2008). Quality characteristics of *Sulgidduk* prepared with Ulmus cortex powder. Korean J. Food Cook. Sci..

[39] Kim MR, Kim MH, Han YS (2021). Antioxidant activities and quality characteristics of *Sulgidduk* added with black carrot (*Daucus carota*L.ssp. sativus var. *atrorubens Alef*.). Korean J. Food Nutr..

[40] Sajilata MG, Singhal RS, Kulkarni PR (2006). Resistant starch-a review. Compr. Rev. Food Sci. Food Saf..

[41] Dundar AN, Gocmen D (2013). Effects of autoclaving temperature and storing time on resistant starch formation and its functional and physicochemical properties. Carbohydr. Polym..

[42] Khawas P, Deka SC (2017). Effect of modified resistant starch of culinary banana on physicochemical, functional, morphological, diffraction, and thermal properties. Int. J. Food Prop..

